# Fibronectin Matrix Polymerization Regulates Smooth Muscle Cell Phenotype through a Rac1 Dependent Mechanism

**DOI:** 10.1371/journal.pone.0094988

**Published:** 2014-04-21

**Authors:** Feng Shi, Xiaochun Long, Allison Hendershot, Joseph M. Miano, Jane Sottile

**Affiliations:** Aab Cardiovascular Research Institute, University of Rochester School of Medicine and Dentistry, Rochester, New York, United States of America; William Harvey Research Institute, Barts and The London School of Medicine and Dentistry, Queen Mary University of London, United Kingdom

## Abstract

Smooth muscle cells are maintained in a differentiated state in the vessel wall, but can be modulated to a synthetic phenotype following injury. Smooth muscle phenotypic modulation is thought to play an important role in the pathology of vascular occlusive diseases. Phenotypically modulated smooth muscle cells exhibit increased proliferative and migratory properties that accompany the downregulation of smooth muscle cell marker proteins. Extracellular matrix proteins, including fibronectin, can regulate the smooth muscle phenotype when used as adhesive substrates. However, cells produce and organize a 3-dimensional fibrillar extracellular matrix, which can affect cell behavior in distinct ways from the protomeric 2-dimensional matrix proteins that are used as adhesive substrates. We previously showed that the deposition/polymerization of fibronectin into the extracellular matrix can regulate the deposition and organization of other extracellular matrix molecules in vitro. Further, our published data show that the presence of a fibronectin polymerization inhibitor results in increased expression of smooth muscle cell differentiation proteins and inhibits vascular remodeling in vivo. In this manuscript, we used an in vitro cell culture system to determine the mechanism by which fibronectin polymerization affects smooth muscle phenotypic modulation. Our data show that fibronectin polymerization decreases the mRNA levels of multiple smooth muscle differentiation genes, and downregulates the levels of smooth muscle α-actin and calponin proteins by a Rac1-dependent mechanism. The expression of smooth muscle genes is transcriptionally regulated by fibronectin polymerization, as evidenced by the increased activity of luciferase reporter constructs in the presence of a fibronectin polymerization inhibitor. Fibronectin polymerization also promotes smooth muscle cell growth, and decreases the levels of actin stress fibers. These data define a Rac1-dependent pathway wherein fibronectin polymerization promotes the SMC synthetic phenotype by modulating the expression of smooth muscle cell differentiation proteins.

## Introduction

Cell differentiation plays an important role during development, tissue repair [1]–[5], and in certain disease pathologies [6]–[9]. Cell differentiation is regulated, in part, by extracellular matrix (ECM) molecules [10]–[13]. Fibronectin has been reported to promote de-differentiation in certain cell types, such as smooth muscle cells (SMCs), mammary epithelial cells, and keratinocytes, and a differentiated phenotype in other cell types, such as osteoblasts and megakaryocytes [10], [11], [14], [15]. Most studies that have examined the effect of ECM molecules on cell differentiation have examined the effect of ECM-coated dishes on cell phenotype. However, much evidence has shown that the fibrillar form of ECM proteins can have effects that are distinct from protomeric proteins [16]–[24]. Fibronectin is produced as a soluble protein, and is assembled/polymerized into fibrillar structures in the ECM by a cell-dependent process [25], [26]. The fibrillar form of fibronectin is believed to be the major functional form of fibronectin in vivo. Hence, examination of the effects of fibrillar fibronectin on cell phenotype are particularly relevant to the in vivo situation.

Smooth muscle cells transition from a differentiated to a synthetic phenotype in response to injury or disease [7], [27]. This modulation of SMC phenotype is thought to be an important contributor to intima-media thickening following arterial injury [27], [28]. Emerging evidence also suggests that SMC phenotypic modulation may play a role in atherosclerosis [9], [29]. Adhesion of SMC to fibronectin-coated dishes has been shown to promote the SMC synthetic phenotype [11], [30]. In contrast, cell adhesion to certain splice variants of fibronectin has been shown to promote SM α-actin expression in some cell types [31]. However, mice lacking these splice variants showed only modest alterations in the SMC differentiated phenotype [32]. We recently showed that a peptide that blocks fibronectin matrix deposition attenuates intimal medial thickening and inhibits the transient decrease in SMC differentiation markers in a flow induced vascular remodeling model [33]. These data suggest that fibronectin deposition into the ECM promotes SMC phenotypic modulation, and provide the first evidence for an in vivo role of fibronectin polymerization in controlling SMC phenotype. To explore the mechanism by which fibronectin polymerization regulates SMC phenotypic modulation, we established an in vitro culture system. Our data show that addition of fibronectin polymerization inhibitors to SMC enhances the differentiated phenotype by regulating serum response factor (SRF)-dependent gene transcription. Further, the ability of fibronectin to regulate the levels of SMC marker proteins occurs by a Rac1 dependent pathway. These data are the first to demonstrate a role for fibronectin polymerization in regulating gene transcription, and define a fibronectin and Rac1 dependent signaling pathway that controls SMC phenotypic modulation.

## Materials and Methods

### Immunological Reagents, Chemicals and Proteins

Antibody to smooth muscle calponin (CALP) was from Dako (Carpinteria, CA); antibodies to SM α-actin (1A4) and mouse antibody to tubulin (DM1A) were from Sigma (St. Louis, MO); rabbit antibody to tubulin and antibodies to phosphorylated p42/44 (pERK), p42/44 (ERK), phosphorylated ELK, and ELK were from Cell Signaling (Danvers, MA); antibody to Rac1 (clone 102) was from BD Transduction Labs (San Jose, CA). Monoclonal antibody L8 to fibronectin, which inhibits fibronectin polymerization, was a gift from Dr. Chernousov [34]. Polyclonal antibody to fibronectin was prepared by immunizing rabbits with mouse fibronectin (ProteinTech, Chicago, IL). IgG was affinity purified from anti-sera on a fibronectin-agarose column. AlexaFluor488 conjugated goat anti-rabbit IgG was from Invitrogen (Invitrogen/Life Technologies, Grand Island, NY). pUR4 (also known as FUD, functional upstream domain) and the control III-11C peptides [33], [35] were purified from bacterial lysates on nickel-agarose columns as described [35]. pUR4 binds to fibronectin, and blocks fibronectin polymerization by inhibiting the binding of soluble fibronectin to the cell surface [33], [35]. Although integrins are important for fibronectin polymerization, pUR4 does not inhibit cell adhesion to fibronectin [33], [35] or collagen (data not shown).

### Cell Culture

Rat aortic SMCs were purchased from Cell Applications (San Diego, CA), and used between passages 3 and 5. For experiments, cells were cultured in Medium 231 (Invitrogen) containing Smooth Muscle Growth Supplement (Invitrogen). 30–60 min after seeding, pUR4B or III-11C were added to a final concentration of 250–500 nM. Monoclonal antibody L8 and control IgG were added at 50 µg/mL. Cells were harvested at various times after treatment. For 5 day experiments, fresh media containing pUR4B, III-11C, L8, or IgG was added on day 3.

### Western blotting

Cells lysates were prepared as described [36] using 1% triton X-100/150 mM NaCl/50 mM Tris, pH 7.4. The lysis buffer was supplemented with complete protease inhibitor cocktail tablet (Roche Applied Sciences, Indianapolis, IN) and 50 mM sodium vanadate. Cell lysates were quantitated using a BCA Protein Assay kit (Thermo Scientific Pierce, Rockford, IL) as per the manufacturer's instructions. Equal amounts of proteins were analyzed under reducing conditions by sodium dodecyl sulfate polyacrylamide gel electrophoresis (SDS PAGE), transferred to PVDF membrane, and probed with the indicated antibodies. In some experiments, blots were also probed with antibodies that recognize tubulin to ensure equal protein loading. Blots were imaged and analyzed using an Odyssey Infrared Imaging System (Li-Cor Biosciences, Lincoln, NE).

### Cell Proliferation

Cells were seeded at 1.5×10^4^ cells/well in 24-well tissue culture dishes in Medium 231 supplemented with smooth muscle growth supplement. 60–90 min after seeding, pUR4B or III-11C were added to a final concentration of 250 nM. The cells were allowed to grow for various lengths of time at 37°C. Fresh media containing inhibitors was added on day 3 and 5. At the indicated times, cells were washed with PBS, then fixed with 1% paraformaldehyde for 30 min at room temperature. Cells were stained with 0.5% Crystal Violet, and the absorbance determined on a spectrophotometer as described [19].

### Immunofluorescence

Immunostaining was performed as described [36]. Fibronectin fibrils were visualized using a polyclonal antibody to fibronectin followed by AlexaFluor488 conjugated goat anti-rabbit IgG. Cells were examined using an Olympus microscope equipped with epifluorescence.

### Quantitative reverse-transcription polymerase chain reaction (qRT-PCR)

mRNA was isolated from SMCs 3–5 days after incubation with 250 nM pUR4 or III-11C control peptides using the mRNeasy kit according to the manufacturer's instructions (Qiagen, Valencia, CA). cDNA was prepared using iScript cDNA synthesis kit (Bio-Rad, Hercules, CA) as per the manufacturer's instructions. SYBR green probe chemistry was used according to the manufacturer's instructions. qRT-PCR reactions were run in triplicate for each sample using a Bio-Rad MyIQ PCR detection system. The data was normalized to the levels of GAPDH in each sample, and the results were averaged. Each experiment was performed ≥3X.

### Luciferase Assays

SMCs were transfected with luciferase reporter constructs using a Gene Pulser electroporator (Bio-Rad) as described [37]. To correct for differences in transfection efficiency, a CMV-β-gal reporter gene was included as an internal control. Cells were seeded in triplicate into 24-well plates. 1 h after seeding, fresh media containing 500 nM pUR4 or III-11C peptides was added. Cell lysates were prepared 3–5 days post transfection, using Passive Lysis Buffer as per the manufacturer's instructions (Promega). Luciferase assays were performed using a Luciferase Assay System according to the manufacturer's instructions (Promega). LacZ levels were assayed using a FluoReporter lacZ/Galactosidase Quantitation Kit (F-2905) according to the manufacturer's instructions (Molecular Probes). Data were analyzed using a FLUOstar OPTIMA plate reader (BMG Labtech, Cary, NC) and expressed as the normalized-fold increase over controls ± s.d. All experiments were performed ≥3X.

### Adenoviruses

Dominant negative (DN) Rho (N19RhoA) and Rac (N17Rac1) were kind gifts from Dr. Anne Ridley [38]. Preparation and transduction of adenoviruses were performed as described previously [39], [40]. Expression of DN Rho and Rac was verified by immunoblotting using an anti-myc antibody (Cell Signaling) that recognizes the myc-tag on the expressed proteins. We previously demonstrated the efficacy of the DN Rho adenovirus [41].

### Rac activity assay

Cell lysates were prepared and immediately mixed with PAK-PDB agarose (Cytoskeleton, Denver, CO) according to the manufacturer's instructions. After washing, proteins were eluted from agarose in SDS PAGE sample buffer and analyzed by western blotting using an antibody to Rac1. A portion of the cell lysate was also analyzed without fractionation.

### Statistical analysis

Data are presented as the mean ± s.e.m. or s.d. Comparisons were made with an unpaired, 2-tailed Student's t test. A difference between the means was considered significant when p<0.05.

## Results

**Figure 1:**
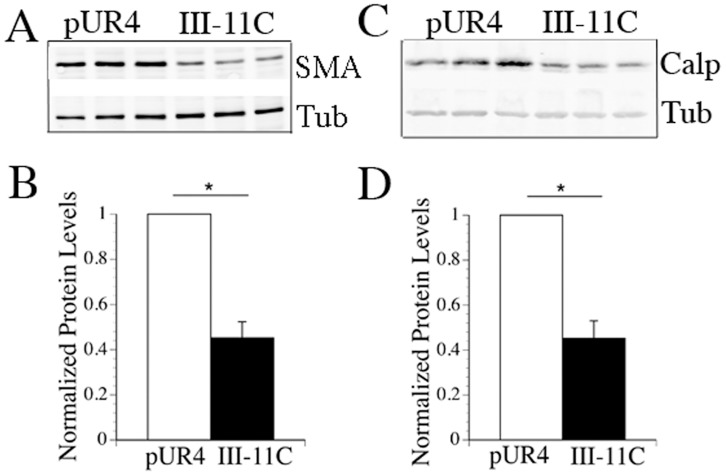
The pUR4 fibronectin inhibitor promotes the SMC differentiated phenotype. Rat aortic SMC were incubated in Medium 231 supplemented with smooth muscle growth supplement. 30–60 min after seeding, pUR4 or the control III-11C peptides were added as described in Methods. Cell lysates were prepared 5 days after seeding. Equal amounts of protein were analyzed by western blotting using antibodies to SM α-actin (A) and calponin (C). Blots were also probed with anti-tubulin antibodies to ensure equal protein loading. The levels of SM α-actin (B) and SM calponin (D) were quantitated using an Odyssey Infrared Imager. Data represent the mean of 3 separate experiments. The levels of SM α-actin and calponin were normalized to the levels of tubulin in each sample; pUR4 treated cells were set equal to 1. The error bar represents the s.e.m. *p<.05.

We previously showed that a peptide that inhibits fibronectin polymerization, pUR4, blocks the transient down- regulation of smooth muscle differentiation markers in the vessel wall during flow induced vascular remodeling [33]. To determine the mechanism by which fibronectin polymerization regulates SMC phenotypic modulation, we developed an in vitro model system in which rat aortic SMC were cultured in growth media containing or lacking the pUR4 fibronectin inhibitor. As expected, the pUR4 inhibitor blocked the formation of fibronectin matrix fibrils (Fig. S1). As shown in Fig. 1, the levels of both smooth muscle alpha actin (SM α-actin) and smooth muscle calponin were 2.3× higher in cells cultured in the presence of the pUR4 inhibitor for 5 days in comparison to control (III-11C) peptide treated cells. Similar results were found when cell lysates were prepared from cells following 3 days of pUR4 treatment (data not shown). Incubation of cells with a different fibronectin polymerization inhibitor, the monoclonal antibody L8, also resulted in higher levels of SM α-actin and calponin in comparison to control antibody treated cells (Fig. S2).

**Figure 2:**
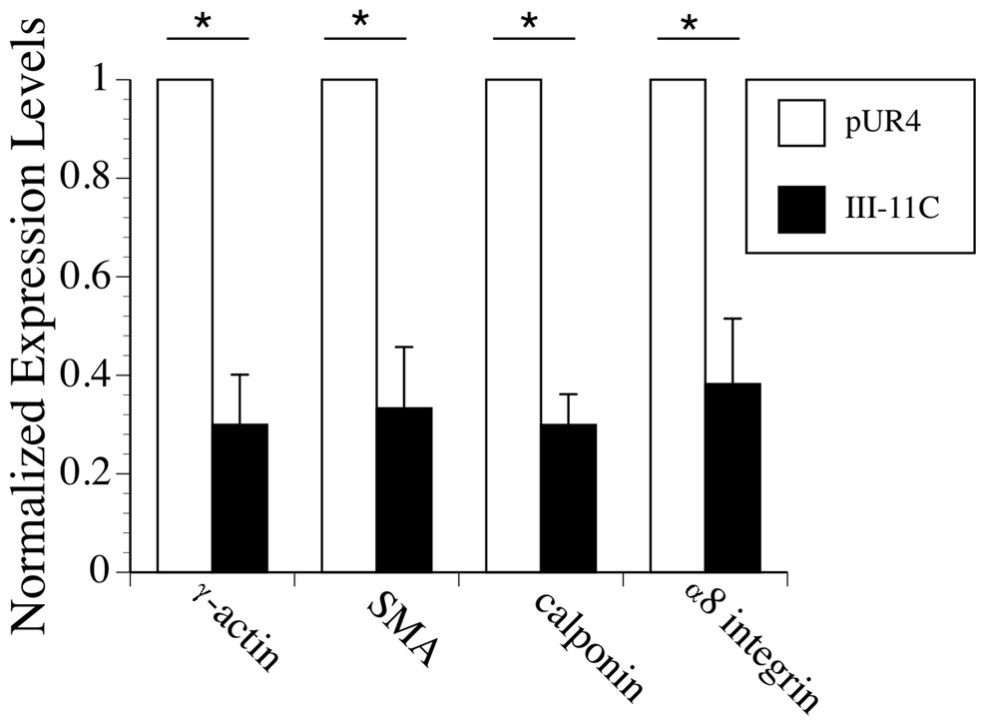
The pUR4 fibronectin inhibitor promotes elevated expression of SM differentiation genes. SMC were incubated in Medium 231 supplemented with smooth muscle growth supplement. 30–60 min after seeding, pUR4 or III-11C were added as described in Methods. RNA was prepared from cells 5 days after seeding as described in Methods. qRT-PCR was performed in triplicate for each sample using SYBR green chemistry as described in Methods. The data was normalized to the levels of GAPDH in each sample. Data represent the average of 4 separate experiments. The relative levels of γ-actin, SM α-actin, calponin, and α8 integrin are shown; pUR4 treated samples were set equal to 1. The error bars represent the s.e.m. *p<.05.

To determine whether fibronectin polymerization similarly regulates mRNA levels of SM α-actin and calponin, we performed quantitative reverse transcriptase polymerase chain reaction (qRT-PCR). As shown in Fig. 2, the presence of the pUR4 fibronectin inhibitor resulted in a 3× increase in SM α-actin and calponin mRNA in comparison to control peptide treated cells. To determine whether other genes that are part of the smooth muscle differentiation program [42] are similarly affected, we quantitated the effect of pUR4 treatment on the levels of smooth muscle γ-actin and α8 integrin. Inhibition of fibronectin polymerization resulted in a 3.3× increase in smooth muscle γ-actin and a 2.6× fold increase in α8 integrin. Hence, the presence of a fibronectin polymerization inhibitor results in increased mRNA and protein levels of multiple smooth muscle marker genes/proteins.

**Figure 3:**
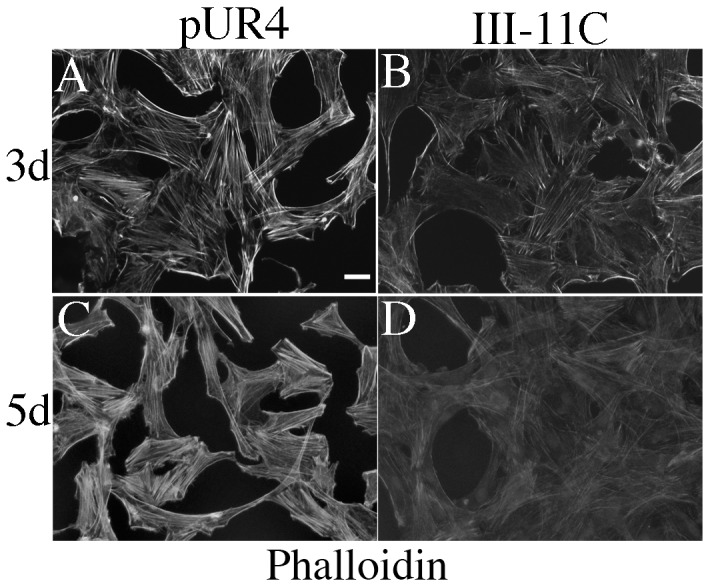
Fibronectin polymerization regulates the organization of actin containing stress fibers. SMC were incubated in Medium 231 supplemented with smooth muscle growth supplement for 3 or 5 days. 30–60 min after seeding, pUR4 or III-11C were added as described in Methods. Actin stress fibers were visualized with rhodamine phalloidin. Bar = 20 µm. The data in panels A and B and those in panels C and D were from separate experiments. Hence, the levels of polymerized actin cannot be directly compared between days 3 and 5.

Many of the genes whose expression are upregulated in differentiated SMC encode cytoskeletal proteins, or proteins that regulate the cytoskeleton. A hallmark of the contractile SMC phenotype is the presence of prominent actin-containing stress fibers. Hence, we asked whether pUR4 treatment altered the abundance of actin-containing stress fibers in SMC. As shown in Fig. 3, SMC treated with the pUR4 peptide for 3 or 5 days showed prominent actin-containing stress fibers (panels A and C) in comparison with control peptide treated cells (panels B and D).

**Figure 4:**
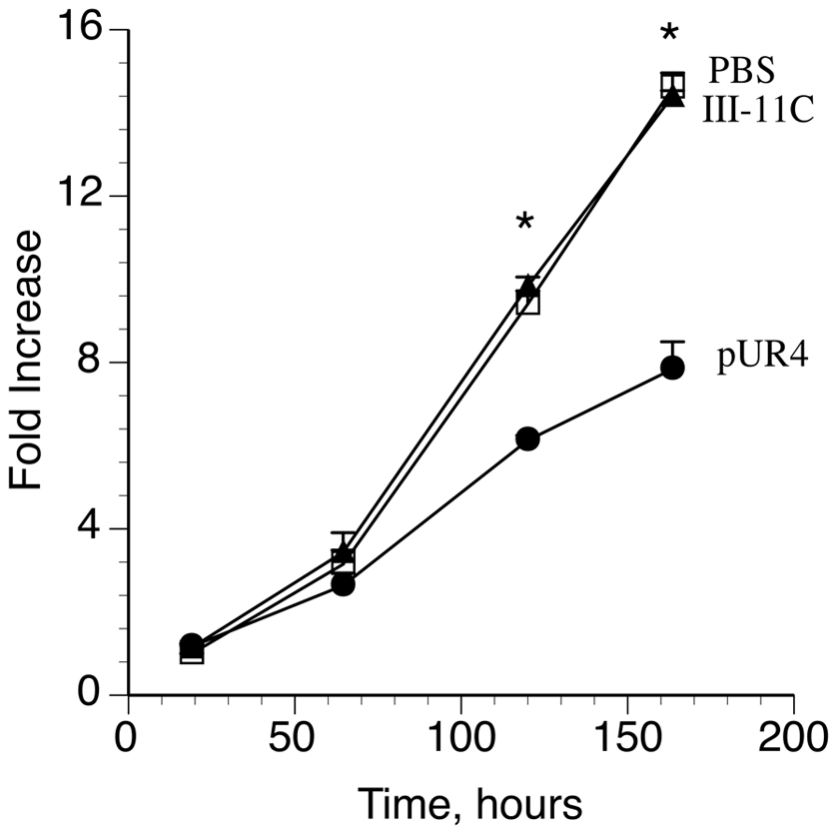
The pUR4 fibronectin polymerization inhibitor attenuates SMC proliferation. SMC were incubated in in Medium 231 supplemented with smooth muscle growth supplement for up to 7 days. 30–60 min after seeding, pUR4 (•), III-11C (▴), or an equivalent volume of PBS (□) were added as described in Methods. Cells were incubated for the indicated time, then washed, fixed with 1% paraformaldehyde, stained with 0.5% crystal violet and the absorbance at 540 nm determined. The absorbance of PBS treated cells 24 h post seeding was set equal to 1. Data represents the mean of 3 separate experiments, and the error bars the s.e.m. *p<.05.

The synthetic SMC phenotype is characterized not only by a reduction in the expression of smooth muscle marker proteins but also by increased cell proliferation. Consistent with these data, the presence of pUR4 significantly blunted cell proliferation in comparison with control peptide treated cells (Fig. 4).

**Figure 5:**
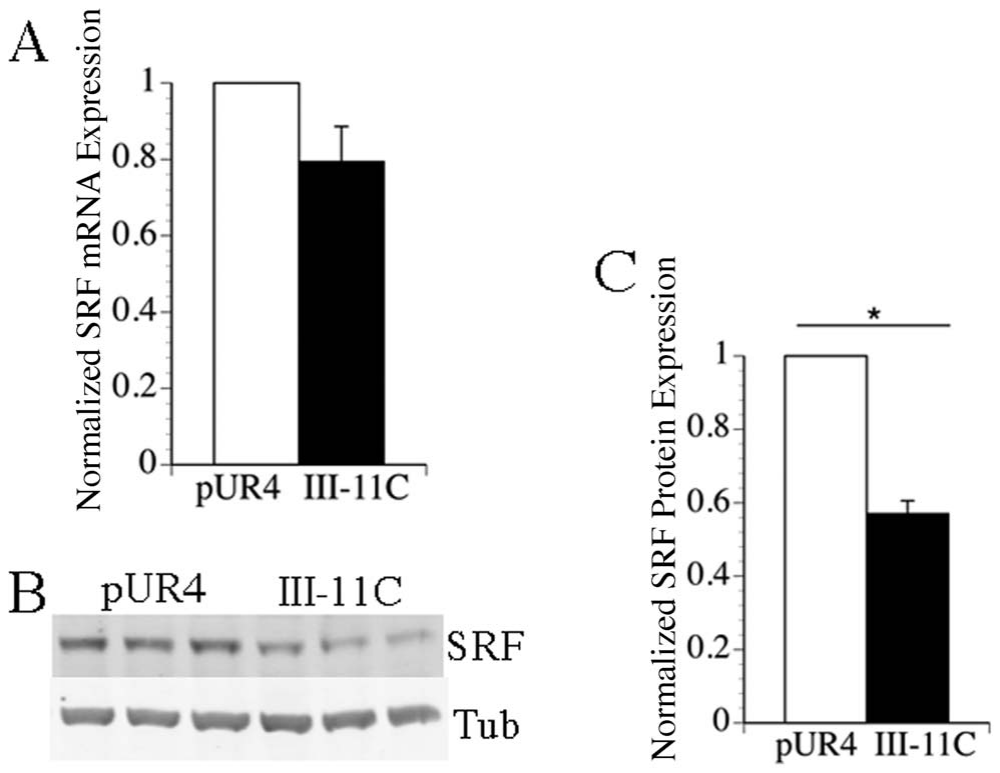
The pUR4 fibronectin inhibitor promotes elevated levels of SRF protein. mRNA or protein were isolated from cells 5 days after seeding as described in Methods. A) qRT-PCR was performed in triplicate for each sample using SYBR green chemistry as described in Methods. The data was normalized to the levels of GAPDH in each sample. Data represent the average of 4 separate experiments, and the error bars the s.e.m. The relative levels of SRF mRNA are shown; pUR4 treated samples were set equal to 1. B) Western blotting was performed using antibodies to SRF. C) SRF protein levels in 3 separate experiments were quantitated using an Odyssey Infrared Imager following normalization to the levels of tubulin in each sample. Normalized SRF levels in pUR4 treated cells were set equal to 1. The error bar represents the s.e.m. *p<.05.

The expression of smooth muscle marker genes is regulated by the transcription factor serum response factor (SRF) in concert with myocardin [7], [28], [43]–[45]. To determine whether the levels of SRF are regulated by fibronectin polymerization, we tested the effect of the pUR4 fibronectin polymerization inhibitor on SRF mRNA and protein levels. Treatment of SMC with pUR4 did not alter the levels of SRF mRNA (Fig. 5A). However, pUR4 treatment resulted in a significant increase in the levels of SRF protein in comparison to control peptide treated cells (Fig. 5B, 5C). These data suggest that one mechanism by which fibronectin polymerization regulates the SMC phenotype is by controlling the levels of SRF.

**Figure 6:**
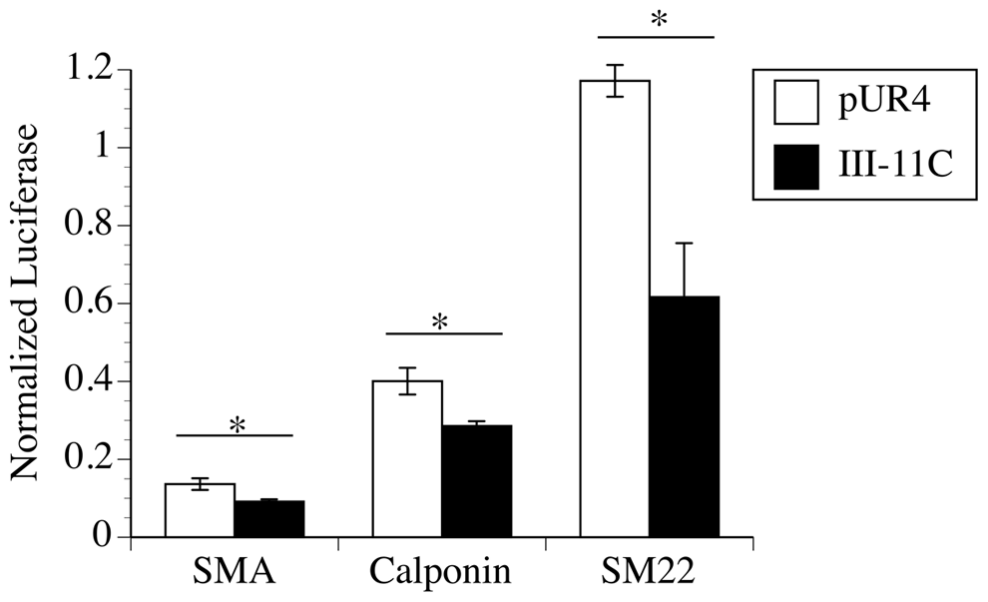
The pUR4 fibronectin polymerization inhibitor enhances the expression of luciferase reporter constructs. SMC were transfected with luciferase reporter constructs containing the promoter/enhancer region of SM αactin, calponin, or SM22. Cells were co-transfected with a β-gal reporter gene. Cells were processed as described in methods, and the levels of luciferase quantitated using a FLUOstar OPTIMA plate reader. Luciferase levels were normalized to Lac Z levels. Data represents the mean of 3 replicate samples, and error bars the s.d. *p<.05. This experiment was repeated 4 times with similar results.

SRF is a transcriptional regulator that binds to CArG elements in the promoter/enhancer region of a variety of genes [42], [46]. Hence, we next asked whether fibronectin polymerization could regulate the transcription of luciferase reporter constructs expressing the CArG-containing promoter/enhancer regions of SM α-actin, calponin, or SM22. As shown in Fig. 6, cells treated with the pUR4 fibronectin inhibitory peptide showed increased luciferase activity in the presence of all 3 reporter constructs in comparison with control peptide treated cells. Luciferase activity was abolished when the CArG elements within the SM α-actin promoter were mutated (data not shown). These data suggest that fibronectin polymerization regulates the SMC differentiation program by regulating the transcription of SRF-dependent genes in a CArG-dependent manner.

**Figure 7:**
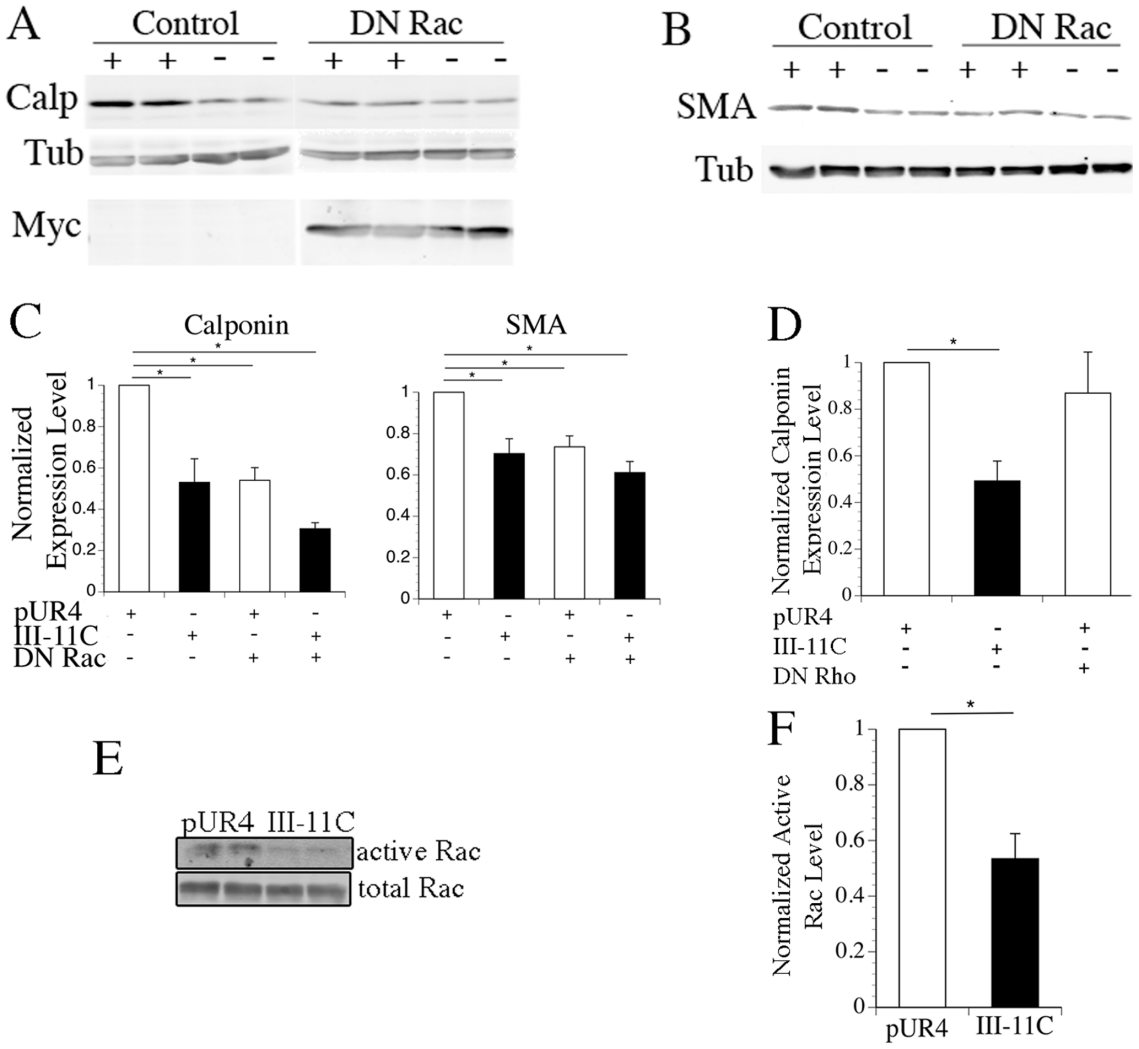
Dominant negative Rac inhibits the ability of the pUR4 fibronectin inhibitor to maintain the smooth muscle differentiated phenotype. SMC were transduced with adenoviruses expressing dominant negative Rac1 (A,B,C), dominant negative RhoA (D), or control adenovirus (A–D). Expression of DN Rac1 (A) and DN RhoA (data not shown) were assessed by immunoblotting with an antibody to myc. Cells were incubated in the presence (+) or absence (−) of pUR4 or control peptides for 5 days. The levels of calponin (A) and SM α-actin (B) were assessed by western blotting, normalized to the levels of tubulin in each sample, and quantitated using an Odyssey Infrared Imager. pUR4 treated cells were set equal to 1. Data represents the mean of 4 separate experiments. The levels of calponin in DN RhoA treated cells were not significantly different from pUR4 treated cells (D). The levels of active Rac were determined in SMC incubated with pUR4 or III-11C peptides (E,F). A representative blot is shown in E, and quantitative data from 3 separate experiments is shown in F. The error bar represents the s.e.m. *p<.05.

Cell adhesion to fibronectin results in a transient increase in the expression of active (GTP-bound) Rho and Rac [47]–[49]. Further, Rho is known to promote fibronectin polymerization [50], [51], suggesting that fibronectin and Rho can reciprocally regulate each other. Rho can also promote the smooth muscle cell differentiated phenotype by regulating the nuclear translocation of the SRF co-factor myocardin related transcription factor (MRTF) [52]–[54]. Rac1 has also been reported to regulate the transcriptional activity of SRF in fibroblasts [53]. Hence, we tested whether Rho or Rac could regulate the ability of fibronectin to modulate SMC phenotype. To do this, SMC treated with pUR4 or control peptides were incubated with adenoviruses expressing dominant negative (DN) RhoA or Rac1, and the effect on expression of SM α-actin and calponin was determined. Unexpectedly, DN Rho did not significantly affect fibronectin-dependent SMC phenotypic modulation (Fig. 7D). However, incubation of cells with DN Rac1 abolished the ability of the pUR4 fibronectin inhibitor to promote the SMC differentiated phenotype (Fig. 7A–7C). Further, treatment of SMC with the pUR4 fibronectin inhibitor resulted in increased levels of active Rac in comparison to control peptide treated cells (Fig. 7E, 7F). These data show that fibronectin polymerization regulates SMC differentiation, in part, by regulating the levels of active Rac1.

SRF can promote the transcription of genes involved in stimulating cell growth. It has been proposed that there is a binary switch wherein growth promoting signals that lead to activation of ERK and ELK-1 promote the dissociation of myocardin-SRF complexes and the formation of SRF-ELK-1 complexes, which leads to the downregulation of the SMC differentiation program and upregulation of genes involved in cell growth [55], [56]. Cell adhesion to fibronectin is known to promote sustained ERK activation and cell growth [57]–[59]. However, the role of fibronectin polymerization in regulating ERK activity is not known. Our data (Fig. 4) as well as published data [18], [19], [60], [61] show that fibronectin polymerization can promote cell proliferation. Hence, we tested whether the presence of ECM fibronectin could stimulate ERK activity. Our data show that the levels of active ERK were similar in cells incubated with the pUR4 fibronectin inhibitor or the control peptide for 4 h, 12 h, 3 days (data not shown) or 5 days (Fig. S3). Consistent with these data, SMC treated with pUR4 contained similar levels of c-fos mRNA in comparison with control peptide treated cells (data not shown). Hence, our data show that fibronectin polymerization regulates SRF-dependent smooth muscle differentiation, but does not regulate the ERK/ELK-1 pathway.

## Discussion

In this manuscript we show that the fibronectin polymerization inhibitor, pUR4, promotes the SMC differentiated phenotype in cultured rat aortic SMC. These data are consistent with our in vivo data demonstrating that periadventitial delivery of pUR4 blocks the transient decrease in expression of SMC marker proteins following flow induced vascular remodeling [33]. pUR4 blocks the ability of fibronectin to promote SMC phenotypic modulation to a “synthetic” phenotype, as evidenced by its ability to increase the expression of SMC marker genes/proteins (Figs. 1 and 2), to inhibit SMC growth (Fig. 4), and to promote the expression of robust actin stress fibers (Fig. 3), which help to maintain a contractile phenotype. pUR4 also blocks the ability of fibronectin to downregulate the levels of SRF protein. Our data also show that the ability of pUR4 to affect the SMC differentiation program depends upon the small GTPase, Rac1 (Fig. 7). These data suggest that fibronectin polymerization regulates SMC differentiation by regulating the expression level of SRF, and by modulating SRF-dependent gene transcription.

The ability of the pUR4 inhibitor to enhance the levels of actin stress fibers (Fig. 3) is consistent with its ability to promote the SMC contractile phenotype (Figs 1 &2 and [33]). However, in other cell types, fibronectin fibrils have been shown to co-align with actin stress fibers [62]–[64] and to promote cell contractility [22], [23]. In addition, pUR4 does not affect actin stress fiber levels in cardiac fibroblasts (data not shown). Hence, the ability of the pUR4 fibronectin polymerization inhibitor to promote actin containing stress fibers may be unique to SMC.

Previous studies have shown that the small GTPase Rho can modulate the SMC differentiation program by regulating actin-dependent nuclear shuttling of the MRTF family of transcription factors [54], [65]. Our data show that DN Rho does not affect fibronectin-dependent SMC phenotypic modulation (Fig 7D). These data are somewhat surprising given the known ability of Rho to modulate SMC differentiation. However, the cells in our study were maintained in serum-containing media. It is possible that the presence of serum masks the effect of Rho on SMC differentiation, since serum can promote MRTF's nuclear localization [54]. Further, in some studies, MRTF has been found to localize to the nucleus of SMC independently of serum or Rho signaling [66]. Interestingly, our data show that Rac1 plays an important role in the ability of fibronectin to modulate SMC differentiation (Fig. 7A–C). The levels of active Rac were upregulated in cells treated with the pUR4 fibronectin inhibitor (Fig. 7E, 7F). Further, the presence of DN Rac1 completely blocked the ability of the pUR4 peptide to promote the SMC differentiation program (Fig. 7A–C). These data show that ongoing fibronectin polymerization is associated with reduced levels of active Rac, and suggest that this decreased Rac activity promotes SMC phenotypic modulation. Previous studies have shown that Rac1 can regulate SRF dependent gene transcription and promote the expression of SM α-actin in fibroblasts [53]. However, to our knowledge, our data are the first to demonstrate that Rac1 can modulate SM α-actin expression and the differentiation program in SMC.

In addition to ECM proteins, integrins have also been shown to regulate gene expression. In some studies, integrins and ECM have been shown to act at the transcriptional level [67]–[70]. However, in other cases, direct demonstration of transcriptional regulation, and the mechanism by which integrins and ECM regulate gene expression have not been elucidated. Further, the effect of fibrillar ECM proteins on cell differentiation and gene transcription has been largely unexplored. Our studies show that fibronectin polymerization promotes the synthetic SMC phenotype by downregulating the transcription of SRF-dependent genes.

SMC phenotypic modulation is associated with decreased expression of SMC marker genes/proteins as well as increased cell proliferation. Our data show that the presence of the pUR4 peptide inhibits SMC proliferation in vitro (Fig. 4). These data are consistent with published data showing that fibronectin polymerization can promote proliferation of multiple cell types [18], [19]. SRF can form complexes with either myocardin, which promotes SMC differentiation, or with Elk1, which promotes cell proliferation. Cell adhesion to fibronectin is known to upregulate the levels of active ERK [57], [71]. However, our data show that fibronectin polymerization does not upregulate the levels of active ERK (Fig. S3) or ElK1 (data not shown), despite the fact that fibronectin was able to promote cell proliferation. It is possible that this is due to the presence of serum in our cell culture system, which could enhance the levels of active ERK.

Our studies define a novel pathway wherein polymerized fibronectin promotes the phenotypic modulation of smooth muscle cells by a mechanism that involves downregulation of SRF dependent gene transcription, and downregulation of the small GTPase, Rac1. The ability of fibronectin polymerization inhibitors to block SMC phenotypic modulation likely contributes to the effectiveness of the pUR4 fibronectin inhibitor in attenuating pathologic vascular remodeling.

## Supporting Information

Figure S1 **The pUR4 fibronectin inhibitor blocks the formation of fibronectin fibrils in SMC.** SMC were grown in Medium 231 supplemented with smooth muscle growth supplement for 5 days. 60 min after seeding, pUR4 or III-11C were added as described in Methods. Cell were fixed, then incubated with a polyclonal antibody to fibronectin. Bar, 20 µm.
(TIF)

Figure S2 **The L8 fibronectin polymerization inhibitor enhances the levels of SM a-actin and calponin.** SMC were incubated in Medium 231 supplemented with smooth muscle growth supplement. 60 min after seeding, 50 µg/mL of antibody L8 or control IgG were added as described in methods. Cell lysates were prepared 5 days after seeding, and western blots probed with antibodies to SM α-actin, calponin, and tubulin.
(TIF)

Figure S3 **Effect of fibronectin polymerization on ERK activity.** SMC were incubated in Medium 231 supplemented with smooth muscle growth supplement. 30–60 min after seeding, pUR4 or III-11C were added as described in Methods. Cell lysates were prepared 5 days after seeding. Equal amounts of protein were analyzed by western blotting using antibodies to phospho ERK (pERK), ERK, or tubulin.
(TIF)
