# Supplementary figures and images for: Fibronectin Matrix Polymerization Regulates Smooth Muscle Cell Phenotype through a Rac1 Dependent Mechanism
Source: PLoS One. 2014 Apr 21;9(4):e94988. doi: 10.1371/journal.pone.0094988 (PMC3994013; doi:10.1371/journal.pone.0094988)

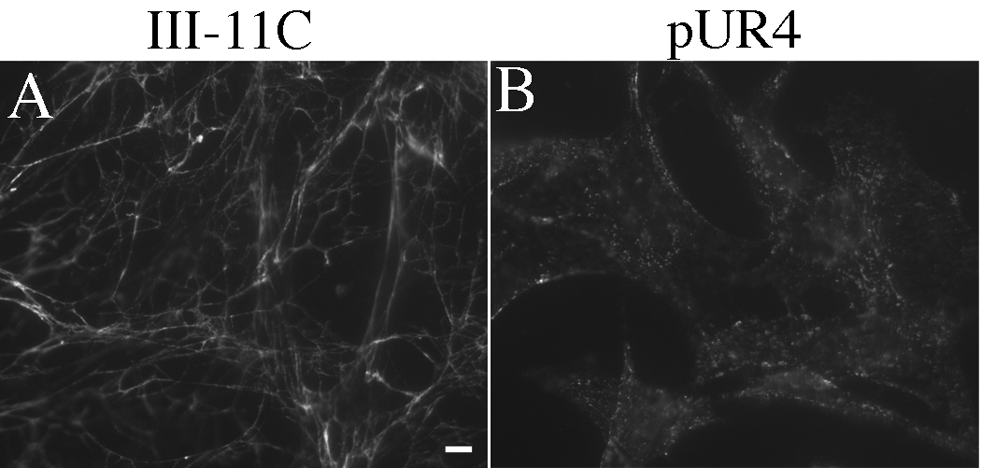

Supplement: Figure S1 — The pUR4 fibronectin inhibitor blocks the formation of fibronectin fibrils in SMC. SMC were grown in Medium 231 supplemented with smooth muscle growth supplement for 5 days. 60 min after seeding, pUR4 or III-11C were added as described in Methods. Cell were fixed, then incubated with a polyclonal antibody to fibronectin. Bar, 20 µm. (TIF) [file pone.0094988.s001.tif]

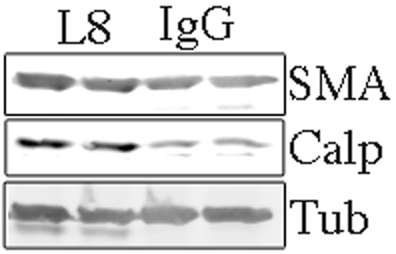

Supplement: Figure S2 — The L8 fibronectin polymerization inhibitor enhances the levels of SM a-actin and calponin. SMC were incubated in Medium 231 supplemented with smooth muscle growth supplement. 60 min after seeding, 50 µg/mL of antibody L8 or control IgG were added as described in methods. Cell lysates were prepared 5 days after seeding, and western blots probed with antibodies to SM α-actin, calponin, and tubulin. (TIF) [file pone.0094988.s002.tif]

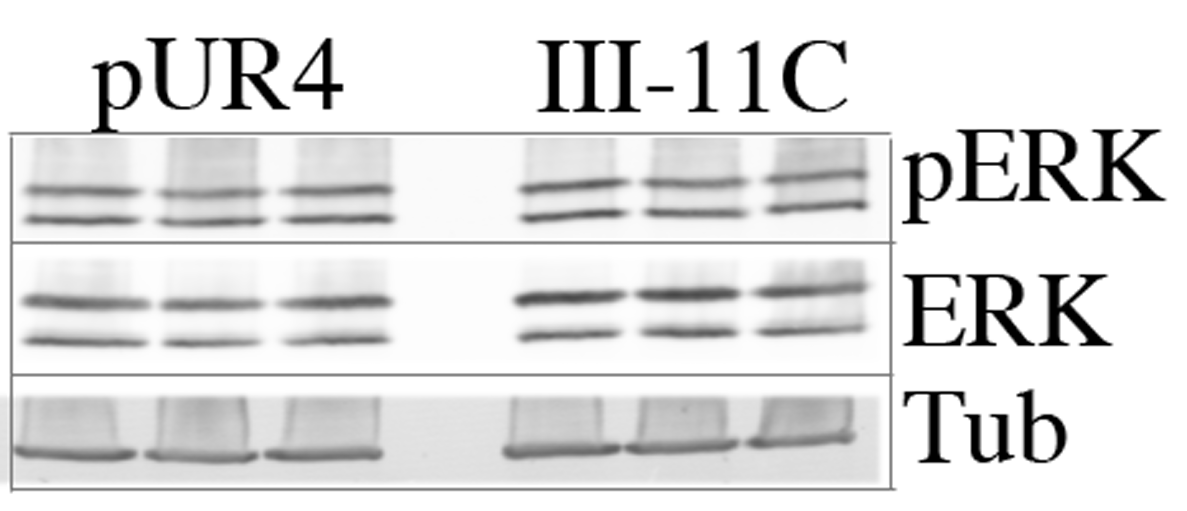

Supplement: Figure S3 — Effect of fibronectin polymerization on ERK activity. SMC were incubated in Medium 231 supplemented with smooth muscle growth supplement. 30–60 min after seeding, pUR4 or III-11C were added as described in Methods. Cell lysates were prepared 5 days after seeding. Equal amounts of protein were analyzed by western blotting using antibodies to phospho ERK (pERK), ERK, or tubulin. (TIF) [file pone.0094988.s003.tif]
